# Improving Patient Education Using Augmented Reality for Spine Fractures: Feasibility Study and Review of Literature

**DOI:** 10.1089/jmxr.2024.0005

**Published:** 2024-04-18

**Authors:** David J. Mazur-Hart, Jamila A. Godil, Brandi W. Pang, Adeline Fecker, Dominic A. Siler, Samantha Yau, James T. Obayashi, Jesse Courtier, Won Hyung A. Ryu

**Affiliations:** 1 Department of Neurological Surgery, Oregon Health & Science University, Portland, Oregon, USA.; 2 Department of Diagnostic Radiology & Biomedical Imaging, University of California San Francisco, San Francisco, California, USA.

**Keywords:** augmented reality, education, fracture, informed consent, spine, trauma

## Abstract

**Purpose:**

The purpose of this prospective feasibility study was to investigate the use of an augmented reality (AR) 3-dimensional (3D) spine fracture model, in an acute inpatient setting on patient education.

**Methods:**

Adult patients (18–89 years) with traumatic spine fractures were included (June 2021–July 2022). Standard spine computed tomography imaging obtained during trauma evaluation was used to create 3D spine models through an open-source application and converted to an AR software application. A 3D-spine model was presented to patients by a resident while discussing spinal anatomy, injury, treatment options, and possible complications. Post consultation, patients completed Likert-scale surveys related to the consultation discussion, with scores subsequently tabulated. Additionally, a review of the literature was performed using the search terms [(virtual reality) AND (patient education)] and [(augmented reality) AND (patient education)] up to May 2023.

**Results:**

Eight patients (50% female), mean age of 57 years (range, 28–85 years), were included. Injuries comprised four cervical, three thoracic, and one lumbar fracture. Treatment(s) included four operative interventions, three braces, and one observation. Median patient Likert-scale score was 36 (range, 33.75–36; maximum = 36). Highest patient scores were for how helpful the 3D spine model was for learning about specific spine anatomy, injury, treatment plan, and possible complications. Seven articles were identified that investigated patient education using 3D mediums. All studies were in the outpatient setting.

**Conclusion:**

AR-based simulation has the potential to improve patient understanding of spinal anatomy, injury, treatment plan, and potential complications over a wide range of traumatic spinal fractures and management plans in the inpatient setting.

## Introduction

Advancement of surgery-related health care technology has led to the development of tools that can assist physicians in providing comprehensive care with improved patient safety. A technological development as applied to neurological surgery is the use of augmented reality (AR). AR is an interactive experience that generates digital content over the real world. In the health care setting, AR can involve medical image (e.g., computed tomography [CT] scan) overlay on a real-world environment (e.g., human body), allowing surgeons to visualize the desired results within context.^1^ This differs from virtual reality (VR), in which an entire world is a digital creation.^2^ The development of compact and powerful AR devices allows such technology to be used in a multitude of environments, which include clinical and educational settings.^3,4^

The use of AR has been shown to be a useful tool in patient and resident education.^5–7^ When a patient is diagnosed with a condition requiring surgical intervention, the onus is on a surgeon to explain the diagnosis and reasoning behind recommendation of either procedural or nonprocedural management. Adequately explaining the course of action has been shown to decrease patient anxiety about the procedure, empower a patient to take ownership of their care, and decrease the cost associated with treatment.^8^

Many factors contribute to a patient’s understanding of their diagnosis and treatment plan, but one of the most impactful factors is health literacy. Providers can support patients by sharing information that is timely, thorough, and without ambiguous medical terminology.^9^ AR provides patients with a three-dimensional (3D) platform to visualize their individual pathology.^10^ The spatial overlay of AR can allow for a better understanding of a topic.^11^

This prospective feasibility study aims to provide the patient experience and the educational role of utilizing an AR 3D spine fracture model in an acute inpatient setting. Our main focus is on postconsultation Likert-scale survey analysis related to a spine treatment consultation discussion. Survey scores were analyzed for understanding of a patient’s particular spine fracture/anatomy, treatment plan, and potential complications. Secondarily, we observed attrition rate through study completion.

## Materials and Methods

### Patients

This is a prospective feasibility study conducted at a single academic institution from October 2021 to July 2022 evaluating adult inpatients admitted with a traumatic spine fracture. Initial evaluation of trauma was performed by the trauma service. Upon discovery of a spine fracture, the spine service was consulted for evaluation and recommendations. All patients were evaluated by the neurological surgery service while on spine call with findings of acute, trauma-induced spine fracture. The neurological surgery service alternates call coverage with the orthopedic surgery service every other week. Inclusion criteria included patients of age 18–89 years who were able to participate in self-care and provide informed consent. In total, 8 patients were included; 15 had consented, but 7 were discharged before completion of the study. The study was approved by the institutional review board (STUDY00022999).

### Augmented reality and 3D spine model

**FIG. 1.**
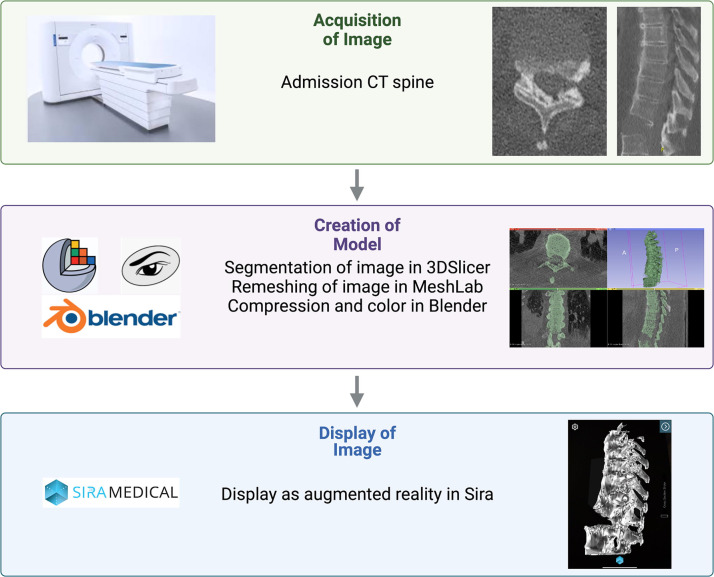
Study workflow from acquisition of images to presentation of augmented reality image.

Patient spine CTs that included the injury location were saved as deidentified DICOM (Digital Imaging and Communications in Medicine) files and uploaded to 3D Slicer (https://www.slicer.org) (Fig. 1). A 3D Slicer software was used to create an initial 3D image as STL (Standard Triangle Language or Standard Tessellation Language) files. This 3D image was segmented/meshed/compressed to AR software using MeshLab (https://www.meshlab.net) and Blender (https://www.blender.org). The final files were uploaded anonymously (to maintain patient confidentiality) to a secure cloud platform, Sira Medical (https://siramedical.com), for final display of the AR images.

Data files were coded to ensure patient-specific images were presented. The AR images were electronically and visually presented using a MERGE Cube (San Antonio, TX). This six-sided cube has the equivalent of a unique QR code on each side. This allows a camera from a mobile device to project an AR image onto the cube, which allows complete circumferential viewing of the model from all angles with tactile feedback to the user.^12^

**FIG. 2.**
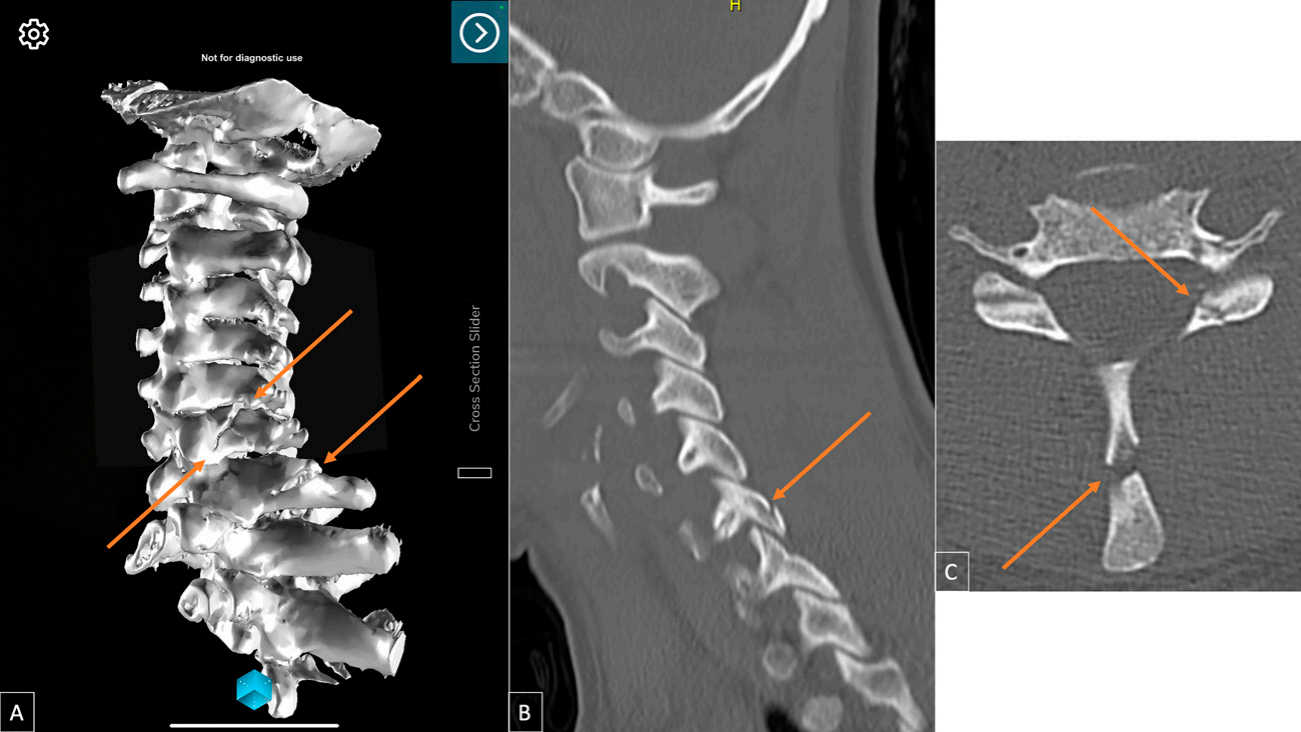
Representative images of a patient’s C6 pedicle/laminar, C6–7 facet, and C7 spinous process traumatic fractures as visualized on **(A)** augmented reality software, **(B)** sagittal computed tomography, and **(C)** axial computed tomography.

This AR image was presented to the patient following the initial clinical consultation. The resident would return with a tablet with the Sira application installed and the MERGE cube. The resident would start by holding the cube while the patient held the tablet. The resident would describe the patient’s anatomy, injury, management plan, and potential complications using AR (Fig. 2). The resident would then give the cube to the patient and hold the tablet for the patient. Following this visit, a Likert-scale survey was given to and completed by the patient.

### Patient survey

The patient survey consisted of eight questions (Table 1). The questions were developed *de novo* as, to the best of our knowledge, no prior study has looked at this patient population. The first four questions focused on patient understanding of their injury, anatomy, proposed treatment plan, and agreement with plan. Responses ranged from 1 (strongly disagree) to 5 (strongly agree). The second set of four questions focused on how helpful the patient found the AR software in learning about their anatomy, injury, proposed plan, and possible complications. These responses included 1 (no help), 2 (mild help), 3 (moderate help), and 4 (great help).

**Table 1.** Patient Survey Questions (Maximum Score = 36)

	Question	Score
1	How would you rate your understanding of the spine injury?	1—Very poor 2—Poor 3—Fair 4—Good 5—Very good
2	I understand where the spine injury is located.	1—Strongly disagree 2—Disagree 3—Neutral 4—Agree 5—Strongly agree
3	I understand why the surgeon chose the treatment plan being offered.	1—Strongly disagree 2—Disagree 3—Neutral 4—Agree 5—Strongly agree
4	I feel comfortable with the surgical plan.	1—Strongly disagree 2—Disagree 3—Neutral 4—Agree 5—Strongly agree
5	How did the 3D model help you to learn about the spine anatomy?	1—No help 2—Mild help 3—Moderate help 4—Great help
6	How did the 3D model help you to learn about the spine injury?	1—No help 2—Mild help 3—Moderate help 4—Great help
7	How did the 3D model help you to understand the proposed treatment/surgery?	1—No help 2—Mild help 3—Moderate help 4—Great help
8	How did the 3D model help you to understand the possible complications related to the proposed treatment/surgery?	1—No help 2—Mild help 3—Moderate help 4—Great help

### Literature review

A literature search of the PubMed/Medline databases was performed using the search terms [(virtual reality) AND (patient education)] and [(augmented reality) AND (patient education)] up to May 2023. We reviewed the search results for studies that used AR/VR in patient understanding/education. We excluded review articles, case reports, and studies that did not include patients, did not include AR/VR models, or did not have institutional access to full articles. Seven articles were identified (Table 2).

**Table 2.** Use of Augmented and Virtual Reality in Preoperative Patient Education since 2015

Author, citation	Modality	Control	Population	Surgical specialty	Improved outcomes	Survey method
Bekelis et al.,^13^	VR	Standard counseling	Adult	Neurosurgery—Cranial and Spine	Anxiety	VAS, EVAN-G, APAIS
Pandrangi,^14^	VR	None	Adult	Vascular	Satisfaction	Likert Scale
Wake et al.,^15^	AR	3D printed, 2D imaging	Adult	General—Prostate	Comprehension	Likert Scale
House et al.,^16^	AR	3D brain model	Adult	Neurosurgery—DBS	Anxiety, Comprehension, Preference	Original questionnaire
Grab et al.,^17^	VR	3D model, standard counseling	Adult	Cardiothoracic	Anxiety, Comprehension, Satisfaction	VAS
Lee et al.,^18^	AR	None	Adult	Neurosurgery—Cranial	Anxiety, Comprehension	Likert Scale
Lo et al.,^19^	AR	Standard counseling	Adult (parents)	Facial Plastics—Cleft Palette	Satisfaction	MERS, VAS, USE

VAS, visual analog scale; DBS, deep brain stimulation; MERS, Mental Effort Rating Scale; USE, usefulness scale; APAPIS, Amsterdam Preoperative Anxiety and Information Scale; EVAN-G, Evaluation du Vécu de l'Anésthésie Génerale.

## Results

### Patient demographics

Eight patients completed the study (Table 3) in the time period described. Their average age was 57 years (range, 28–85 years) and four were female (50%). Injury locations were four cervical, three thoracic, and one lumbar (50%, 37.5%, and 12.5%, respectively). Four patients (50%) experienced a neurological deficit. Four patients (50%) required operative intervention, three (37.5%) bracing, and one (12.5%) did not require any intervention. There were an additional seven patients who consented, but did not finish the study because they were discharged before interaction with the model and completion of the survey. This resulted in a 46.7% attrition rate.

**Table 3.** Patients’ (*n* = 8) Demographics and Survey Scores, with Patient and Resident Scores Compared

Age (years)	Sex (Male/Female)	Injury location	Neurological examination	Treatment	Patient questionnaire median score
71	Male	C6–7 translation	BUE weak	C6–7 ACDF C5–T1 PIF C6–7 laminectomy	36
74	Male	T11–12 extension	Intact	T9–L2 percutaneous fixation	36
28	Female	C6 pedicle/facet/laminar fracture C7 SP fracture	BUE weak	C5–7 ACDF	36
55	Male	L2 burst	LLE weak	TLSO	36
85	Female	C1 burst	Intact	Ccollar	36
56	Male	T11 burst	Baseline LUE/LLE weak	TLSO, as needed	32
46	Female	C2 hangman’s fracture	Intact	Ccollar	36
42	Female	T12 extension	Intact	T10–L2 PIF	33

ACDF, anterior cervical discectomy and fusion; BUE, bilateral upper extremity; Ccollar, cervical collar; LLE, left lower extremity; LUE, left upper extremity; PIF, posterior instrumented fusion; Q, question; SP, spinous process; TLSO, thoracic lumbar sacral orthoses.

### Patient survey

The median patient score was 36 ([33.75–36]; range, 32–36).

**FIG. 3.**
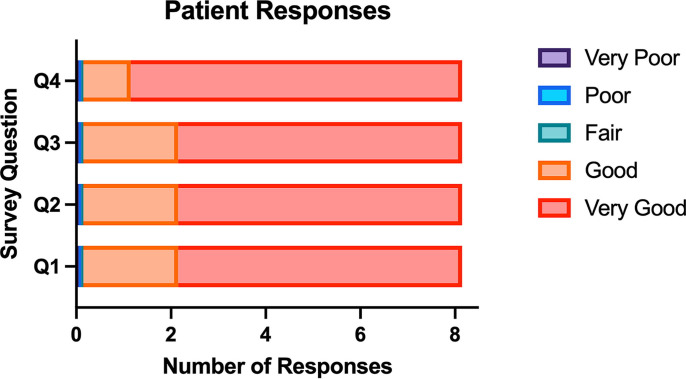
Patient completed Likert survey responses for questions 1–4. These questions represent the patient’s understanding of their injury and plan. Results show overwhelmingly positive responses.

**FIG. 4.**
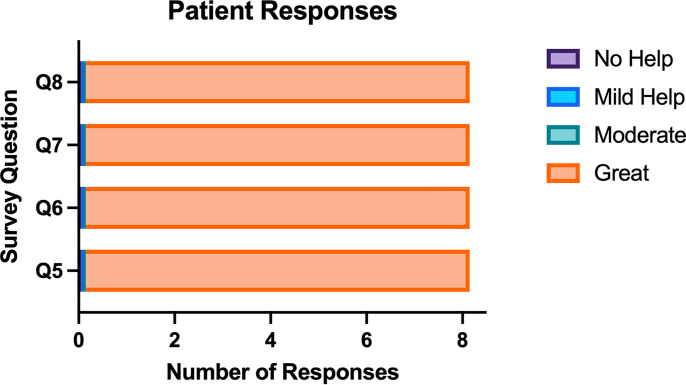
Patient-completed Likert survey responses for questions 5–8. These questions represent how the augmented reality software helped with understanding anatomy, injury, plan, and complications. Results show overwhelmingly positive responses.

The maximum obtainable score was 36. Median responses for each question were as follows: (1) How would you rate your understanding of the spine injury? 5—very good [4.25-5]; (2) I understand where the spine injury is located, 5—very good [4.25-5]; (3) I understand why the surgeon chose the treatment plan being offered, 5—very good [4.25-5]; (4) I feel comfortable with the surgical plan, 5—very good [5-5]; (5) How did the 3D model help you to learn about the spine anatomy? 4—great help [4-4]; (6) How did the 3D model help you to learn about the spine injury? 4—great help [4-4]; (7) How did the 3D model help you to understand the proposed treatment/surgery? 4—great help [4-4]; and (8) How did the 3D model help you to understand the possible complications related to the proposed treatment/surgery? 4—great help [4-4] (Figs. 3 and 4).

All patient scores were the maximum (4—great help) for the AR software’s ability in patients’ understanding of specific anatomy, injury, proposed plan, and possible complications. Cervical and lumbar injury scores were 36. Thoracic injury scores were 32 and 33, with a single 36.

## Discussion

Patient education and understanding of their disease to reach true patient autonomy are difficult to achieve. This is especially the case in a posttraumatic inpatient setting. We aimed to use AR as a technological aid to assist in patient understanding. We found that an AR 3D spine model can be successfully used in the acute inpatient setting for traumatic spine fractures. We initiated the project with no additional staff. We purchased a tablet with a camera to run the application and a MERGE cube for the display of the AR image. Our Likert-scale survey scores show that patients find AR to be of “great help” in learning about their relevant spine anatomy, injury pattern, treatment plan, and possible complications. We also found that the integration of AR does take time and patients may get discharged before interacting with the model.

Studies have consistently shown that the education of patients to involve them in their care produces better patient outcomes.^20–25^ Patient education is especially challenging in surgical fields and in obtaining informed consent.^26,27^ One study used a preoperative informative app to help patients understand postoperative expectations following deep brain stimulation for Parkinson’s disease.^28^ They found this improved patient satisfaction regardless of the motor outcome. Another study looked at patient and parent preferences for implant density before scoliosis surgery.^29^ They found that education regarding this resulted in more decision-makers being willing to enter the trial.

AR is a powerful new technological advancement. This technology has been used for operative planning and intraoperative assistance in tandem with navigation and robotics.^30–39^ A powerful feature of AR is the visualization and conceptualization of 2D screens/images into 3D objects. Interacting with 3D objects allows for greater conceptualization, as seen in a study by Sezer et al., where they randomized students to a mock role of receiving a brain tumor consultation using 2D, 3D, or AR technology.^40^ They found that both the 3D and AR technology aided in memory recall from the mock consultation.

A review of the literature identified seven published studies evaluating the role of AR/VR in patient education (Table 2). A study in vascular surgery used an AR-modeled abdominal aortic aneurysm to teach patients, with survey results showing benefits in comprehension and/or satisfaction.^14^ This was taken a step further in a study of renal and prostate cancers that used patient-specific 3D printed and AR models in the preoperative clinic setting.^15^

Patients found this helpful for their understanding of their diseases and surgical procedure in patients with kidney and prostate cancer. A cardiothoracic surgery study compared paper instructions, VR instructions, and 3D model instructions in preparing patients for surgery.^17^ They found that all three methods improved patient understanding, the VR and 3D model scored higher for quality of education, and the VR reduced anxiety the greatest. A similar study was performed in otolaryngology assessing mental effort and satisfaction comparing leaflets and AR information given to families of patients undergoing cleft lip surgery.^19^ They found that AR had greater ease of use and better satisfaction scores.

There were three studies in the field of neurosurgery, which showed improvements in anxiety and/or comprehension.^13,16,18^ Lee et al. reviewed cranial pathology with patients in clinic with positive survey results for comprehension and satisfaction.^18^ House et al. assessed patients with epilepsy through surveys for comprehension and anxiety using either 3D model or mixed reality tool, with more positive answers seen in mixed reality.^16^ Finally, Bekelis et al. randomized patients getting elective cranial or spinal surgery to receive either a VR experience or routine experience describing their perioperative experience.^13^ Patients found the VR better prepared them for their operative day with less stress and improved satisfaction. All studies were conducted using surveys.

Here, we surveyed patients for how the AR aided in their understanding of the consultation. To the best of our knowledge we present for the first time how AR can improve patient understanding during inpatient consultations. In addition, our study specifically targets spinal injury. We selected traumatic spinal injury, as the spine is difficult to conceptualize in a 3D environment based on CT images alone. AR allows 3D rendering and display for the patient.

To our knowledge, we present for the first time how AR can improve patient-perceived education during spine inpatient consultations. Our Likert survey showed “very good,” the highest possible positive result. The highest possible AR software scores of “very good” were for how AR helped with patient-perceived comprehension of their spinal anatomy, traumatic fracture, proposed plan, and possible complications. Owing to these favorable responses, further development into a study evaluating AR to conventional CT for patient understanding will be pursued.

### Augmented reality limitations

The limitations of the AR technology described includes time spent segmenting images and optimizing them. This will, hopefully, be diminished by a fully automated segmentation and model creation. Timing is especially important in the inpatient setting, where care progresses quickly, as seen by our attrition rate. AR does not allow for appropriate haptic feedback, as the model is projected onto another object, similar to a green screen. There is cost associated with purchasing a device that can run the application software and an object to serve as the AR source. Finally, this technology does not address all issues related to informed consent such as language barriers.

### Study limitations

Study limitations include patient selection bias and feasibility with a small number of included patients (*n* = 8), which limits generalizability of the results. First, patient enrollment was challenging. Significant spinal injury often left the patient unable to provide consent. Moderate spinal trauma often left the patient experiencing altered consciousness because of analgesics. Minor spinal trauma resulted in discharge before enrollment in the study. This resulted in a lower-than-expected enrollment in the study. Second, there was no control group, e.g., educated by exposure to CT alone without AR. No control group was included, as this was a feasibility study, but this means it cannot be compared with conventional studies. Future studies will compare consultation and education with anatomical models and 2D images alone with 3D AR.

Evaluation of AR use for other pathologies (inpatient and outpatient settings) in favor of education purposes is also warranted. Third, prospective measure(s) of patient satisfaction, anxiety, cognitive load, and cost were beyond the scope of this feasibility study. Additionally, survey questions were generated *de novo* for this feasibility study, as no validated questions exist for this study methodology. Finally, no assessment was given to test patient knowledge pre- and post-teaching, as this was outside the scope of the study.

In current practice, no test is offered to patients to ensure true informed consent is obtained as distilling years of education and training to a lay person in medical distress is difficult. This is the inherent reason behind the current study. Future studies looking at patient or resident education may benefit from this. A PubMed literature search was conducted, as it is generally recognized as an acceptable source of clinical information. A broader search may yield additional published sources from journals that are not indexed with PubMed. The authors acknowledge that consultation time with individual patients was not specifically measured (with or without an AR component).

## Conclusion

AR holds a great deal of promise for expanding and improving on clinical and educational information, as applied to neurological surgery.^3,4^ This prospective feasibility study shows that AR can be used in the inpatient setting to help patient-perceived understanding of spinal injury. AR will be important for spine surgeons and patients moving into the future, particularly if applied to complex spine cases.

## Abbreviations Used

2D: 2-Dimensional
3D: 3-Dimensional
ACDF: Anterior Cervical Discectomy and Fusion
APAPIS: Amsterdam Preoperative Anxiety And Information Scale
AR: Augmented Reality
BUE: Bilateral Upper Extremity
Ccollar: Cervical Collar
CT: Computed Tomography
DBS: Deep Brain Stimulation
DICOM: Digital Imaging and Communications in Medicine
EVAN-G: Evaluation du Vècu de l’Anèsthesia Gènerale
LLE: Left Lower Extremity
LUE: Left Upper Extremity
MERS: Mental Effort Rating Scale
PIF: Posterior Instrumented Fusion
Q: Question
SP: Spinous Process
STL: Standard Triangle Language or Standard Tessellation Language
TLSO: Thoracic Lumbar Sacral Orthoses
USE: Usefulness Scale
VAS: Visual Analog Scale
VR: Virtual Reality
